# Utilization of transposable element *mPing* as a novel genetic tool for modification of the stress response in rice

**DOI:** 10.1007/s11032-013-9885-1

**Published:** 2013-06-08

**Authors:** Kanako Yasuda, Makoto Ito, Tomohiko Sugita, Takuji Tsukiyama, Hiroki Saito, Ken Naito, Masayoshi Teraishi, Takatoshi Tanisaka, Yutaka Okumoto

**Affiliations:** 1 Graduate School of Agriculture, Kyoto University, Kitashirakawa, Sakyo-ku, Kyoto, 606-8502 Japan; 2 Genebank, National Institute of Agrobiological Sciences, Kannondai 2-1-2, Tsukuba, Ibaraki 305-8602 Japan

**Keywords:** Rice, Transposable element, Transposition, *mPing*, Gene expression, Reverse genetics

## Abstract

Transposable elements (TEs) are DNA fragments that have the ability to move from one chromosomal location to another. The insertion of TEs into gene-rich regions often affects changes in the expression of neighboring genes. Miniature *Ping* (*mPing*) is an active miniature inverted-repeat TE discovered in the rice genome. It has been found to show exceptionally active transposition in a few *japonica* rice varieties, including Gimbozu, where *mPing* insertion rendered adjacent genes stress-inducible. In the Gimbozu population, it is highly possible that several genes with modified expression profiles are segregating due to the de novo *mPing* insertions. In our study, we utilized a screening system for detecting de novo *mPing* insertions in the upstream region of target genes and evaluated the effect of *mPing* on the stress response of the target genes. Screening for 17 targeted genes revealed five genes with the *mPing* insertion in their promoters. In most cases, the alteration of gene expression was observed under stress conditions, and there was no change in the expression levels of those five genes under normal conditions. These results indicate that the *mPing* insertion can be used as a genetic tool to modify an expression pattern of a target gene under stress conditions without changing the expression profiles of those under natural conditions.

**Electronic supplementary material:**

The online version of this article (doi:10.1007/s11032-013-9885-1) contains supplementary material, which is available to authorized users.

## Introduction

Transposable elements (TEs) are DNA fragments that have the ability to move from one chromosomal location to another. Recent genome projects on several species have revealed that TEs are widespread in all organisms, from bacteria to humans, and constitute a significant portion of eukaryotic genomes (Kazazian 2004). They represent over 60 % of the human genome (de Koning et al. 2011), 35 % of the rice genome (Turcotte et al. 2001), and over 85 % of the maize genome (Schnable et al. 2009). Many kinds of TEs have been identified to date, and some of these have increased their copy number up to several thousand copies per genome (Lander et al. 2001; Khan et al. 2011).

The insertion of TEs into gene-rich regions often changes the expression level of neighboring genes through alteration of the DNA sequence. TE insertion can also sometimes disrupt the original *cis*-elements and occasionally triggers epigenetic silencing, leading to inhibited interaction between the *cis*-element and the *trans*-element (Corces and Geyer 1991; Martin et al. 2009). On the other hand, TEs that exhibit promoter activity are able to transcribe the adjacent intergenic sequence, and TE-derived sequences can also provide new regulatory elements for neighboring genes, resulting in these genes showing novel expression patterns (Kloeckener-Gruissem et al. 1992). These TE-derived genetic changes have been broadly used as a tool in reverse genetics. Consequently, TE tagging mutant panels have been established in rice with endogenous retrotranspon *Tos17* (Miyao et al. 2003) and exogenous elements, such as *Ds* and T-DNA (Kolesnik et al. 2004; Jeon et al. 2000).

Miniature *Ping* (*mPing*) is the active miniature inverted-repeat transposable element (MITE) discovered in the rice genome (Jiang et al. 2003; Kikuchi et al. 2003; Nakazaki et al. 2003). It is a 430-bp DNA fragment that contains 15-bp terminal inverted repeats and it introduces target site duplications of TAA or TTA, typical characteristics of a *tourist*-like MITE family. Transposase, which is necessary for the transposition of *mPing*, is provided from the autonomous elements *Ping* and *Pong*. They contain open reading frame (ORF) 1 and ORF2, both of which are needed for the transposition of *mPing*. ORF1 encodes a protein similar to the DNA-binding domain of the *myb* transcription factor and ORF2 encodes transposase (Jiang et al. 2003; Yang et al. 2007). *mPing*, a deletion derivative of *Ping*, contains the promoter region of ORF1.

The exceptionally active transposition of *mPing* has been observed in a few *japonica* rice varieties, including Gimbozu (Naito et al. 2006). In the Gimbozu genome, the copy number of *mPing* exceeds 1,000 copies, while that of Nipponbare is just 51 copies. Furthermore, *mPing* increases its copy number by approximately 40 per plant per generation (Naito et al. 2006). A detailed investigation of de novo *mPing* insertion sites revealed that *mPing* preferentially inserted in the 1- to- 500-bp upstream regions of genes. The results of this study showed that in fact 9.4 % of de novo insertions of *mPing* were distributed in the 500-bp upstream regions of genes (Naito et al. 2009). These authors also reported that *mPing* contained putative stress-responsive *cis*-elements in its sequence and rendered adjacent genes stress-inducible (Naito et al. 2009).

In the Gimbozu population, it is highly possible that several genes with modified expression profiles caused by the *mPing* insertion are segregating. Therefore, application of the appropriate evaluation system should allow researchers to use *mPing* as a genetic tool to modify the expression of a target gene. Several other active transposable elements of rice are suitable tools for gene tagging systems because of their destructive effects on gene function (Miyao et al. 2003; Tsugane et al. 2006; Takagi et al. 2007; Terada et al. 2007). *mPing* could also be used for the fine-tuning of gene expression. The unique nature of *mPing* makes it well suited to enrich the genetic resources for conventional rice breeding. In this study, we designed a screening system that detects *mPing* insertion in upstream regions of the target genes and we observed the screening efficiency. We also evaluated the effect of *mPing* insertion on the stress response through gene expression analyses under stress conditions.

## Materials and methods

### Plant material

*Oryza sativa* ssp. *japonica* cv. Gimbozu was used. In 2010, 11,520 Gimbozu plants were grown in the experimental field of Kyoto University to establish a DNA pool and a seed pool.

### Construction of DNA pool and seed pool

Approximately 2-cm-long leaf blades were harvested from each plant and divided into eight-plant bulked samples. The bulked samples were crushed using a Multi-Beads Shocker (Yasui Kikai, Osaka, Japan) in extraction buffer (100 mM Tris–HCl pH 8.0, 1 M KCl, and 10 mM EDTA; Yamamoto et al. 2007a). After centrifugation, the supernatant was recovered, and an equal volume of isopropyl alcohol was added. Precipitated DNA was recovered by centrifugation, and the pellet was first washed with 75 % ethanol, then dried and dissolved in 1/10 TE (10 mM Tris–HCl pH 8.0 and 1 mM EDTA). A total of 1,440 DNA bulked samples were diluted to 25 ng/μl per sample, and the samples were applied to fifteen 96-well plates for PCR analysis using gene-specific primers.

At plant maturation, we harvested a single panicle from all of the 11,520 plants. Eight panicles of the plants (corresponding to an eight-plant DNA bulked sample) were stored in a bag. We then made 1,440 seed bags (seed pool) corresponding to each of the 1,440 DNA bulked samples (DNA pool).

### PCR amplification

PCR primers were designed to amplify the approximately 500-bp target regions [Electronic Supplementary Material (ESM) Table 1]. PCR analyses were performed in a total volume of 5 μl, with 25 ng of DNA. DNA was mixed with 2.5 μl of 2× GoTaq^®^ Green Master Mix (Promega, Madison, WI, USA), 0.5 μl of 2.5 μM primers, and 0.25 μl dimethyl sulfoxide. PCR steps for amplification were as follows: heat denaturation at 94 °C for 3 min, 40 cycles of denaturation at 94 °C for 30 s, annealing at 57 °C for 45 s, and extension at 72 °C for 1.5 min. To select the bulked samples that harbor *mPing* in the upstream region of the target gene, PCR products were separated by electrophoresis in a 0.8 % agarose gel.

### Selection of *mPing* insertion mutants with DNA pool and seed pool

**Fig. 1:**
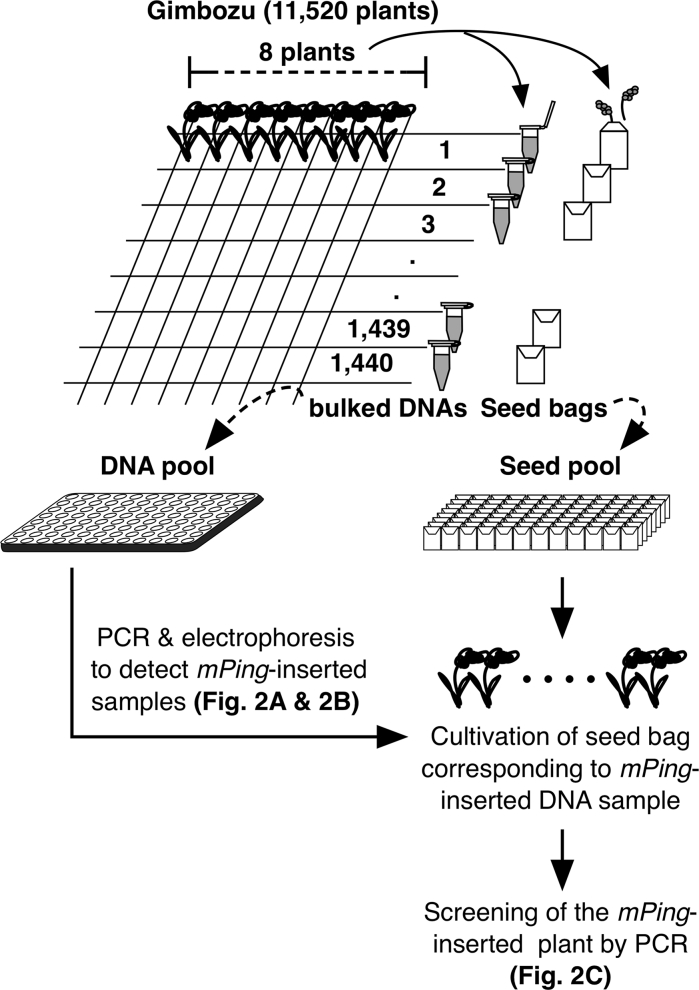
Screening scheme of *mPing*-inserted alleles. A total of 11,520 Gimbozu plants were cultivated and self-fertilized. A single DNA bulked sample and a single seed bag were compiled from eight plants. A set of DNA bulked samples (*DNA pool*) was used for the primary screening by PCR. PCR was performed using target-specific primers (see Fig. 2a, b). Progenies of eight plants in a seed bag corresponding to a DNA bulked sample with *mPing* insertion were cultivated, and DNA was then extracted for the secondary screening with target-specific PCR (see Fig. 2c)

The DNA pool prepared from 11,520 Gimbozu plants was used for the primary screening of *mPing*-inserted alleles that have a new *mPing* insertion in the 1- to 500-bp upstream region from the transcription start site (TSS) of the target genes (Fig. 1). *mPing*-inserted alleles were detected based on the length difference: PCR products with the insertion were approximately 430 bp longer than the original promoter region. Before the screening, we confirmed that the eight-plant bulk sample was small enough to detect a single heterozygous plant for *mPing*-inserted allele through PCR (data not shown). Among the 1,440 DNA bulked samples, 17 were omitted from PCR analysis due to the low quality of the DNA.

In the Gimbozu genome, the number of new insertions of *mPing* is around 40 per plant per generation (Naito et al. 2006). Based on previous results, we assumed that 10 % of new *mPing* insertions would be located at the 5′-flanking regions of genes, especially at up to the 500-bp upstream regions from TSS (Naito et al. 2009). There are ≤30,000 protein coding genes (Itoh et al. 2007). Thus, 4/30,000 is a rough estimation of the probability that a gene newly acquires the *mPing*-inserted promoter per plant per generation. Then, (1–4/30,000)^11,520^ ≈ 0.22 is the probability that a gene is not harboring the *mPing*-inserted promoter in the 11,520 plants. This calculation indicates that we could expect at least one *mPing*-inserted promoter in about 78 % of the rice gene plants in a Gimbozu population of this size.

As shown in Table 1, we selected the stress-related genes as the target genes whose overexpression improved stress tolerance, except *salT* and *wsi18*. The upstream region (−500 bp from TSS) of these genes was amplified using specific primer pairs. When we found a DNA bulked sample that exhibited a fragment that was approximately 430 bp longer than the original fragment, we subjected the seed bag corresponding to this DNA bulked sample to further screening. We cultivated 64 plants (8 plants × 8 lines) and examined their genotypes for an *mPing*-inserted allele. A plant homozygous for an *mPing*-inserted allele and a plant homozygous for a non-inserted allele were selected as a *mPing*+ (plus) plant and an *mPing*− (minus) plant, respectively. Selfed seeds harvested from the *mPing*+ and *mPing*− plants were used to raise *mPing*-inserted lines and non-inserted lines, respectively. The effects of the *mPing* insertion were evaluated by comparing an *mPing*-inserted line and a non-inserted line that originated from a single plant.

**Table 1:** Target genes

Gene	Gene function	Acquired abiotic stress tolerance by overexpression	Reference
*OsDREB1A*	Transcription factor	Salt, drought, and cold tolerance	Ito et al. (2006)
*DREB1D*	Transcription factor	Salt and cold tolerance	Zhang et al. (2009)
*OsDREB1F*	Transcription factor	Salt, drought, and cold tolerance	Wang et al. (2008)
*ZFP252*	Zinc finger, C_2_H_2_-type domain containing protein	Salt and drought tolerance	Xu et al. (2008)
*ZFP182*	Zinc finger, C_2_H_2_-type domain containing protein	Salt tolerance	Huang et al. (2007)
*SNAC1*	Transcription factor	Salt and drought tolerance	Hu et al. (2006)
*OsNAC6*	Transcription factor	Salt and drought tolerance	Nakashima et al. (2007)
*ONAC045*	Transcription factor	Salt and drought tolerance	Zheng et al. (2009)
*OsLEA3*-*1*	Late embryogenesis abundant protein	Drought tolerance	Xiao et al. (2007)
*MYBS3*	Transcription factor	Cold tolerance	Su et al. (2010)
*OsNHX1*	Na+/H+ antiporter	Salt tolerance	Fukuda et al. (2004)
*OsGS2*	Glutamine synthetase	Salt tolerance	Hoshida et al. (2000)
*OsCDPK7*	Calcium-dependent protein kinase	Salt, drought, and cold tolerance	Saijo et al. (2000)
*SalT*	Salt-induced protein	No data	
*wsi18*	Late embryogenesis abundant protein	No data	
*OsWRKY11*	Transcription factor	Drought and heat tolerance	Wu et al. (2009)
*OsbZIP*	Transcription factor	Salt and drought tolerance	Xiang et al. (2008)

### Database search of the *cis*-elements in the upstream region of genes

A database search of the promoter for *OsDREB1A*, *ZFP252*, *ONAC045*, and *OsCDPK7* was performed to look for core promoters [TATA box, pyrimidine patch (Y Patch), and regulatory element group (REG)] using the Plant Promoter Database (PPDB; Yamamoto and Obokata 2008). The function feature of the REG was searched by employing the Plant *cis*-acting Regulatory DNA Elements database (PLACE; Higo et al. 1999). The TATA box and Y Patch are orientation-sensitive factors, while REGs are orientation-insensitive factors that appear upstream of the TATA box (−20 to −400 bp; Yamamoto et al. 2007b). In this analysis, we searched for REGs located up to −800 bp from the TSS because there are no stress-responsive REGs in the region up to −400 bp from the TSS in all target genes.

### Stress treatments

Seeds were sterilized with 1,000-fold diluted fungicide (Benlate^®^; Sumitomo Kagaku, Kogyo, Japan) for 24 h and then immersed in water at 25 °C in the dark. After 72 h of incubation, germinated seeds were cultivated in Kimura B solution in a greenhouse under natural daylight. Seedlings at the three-leaf stage were transferred to a growth chamber that was kept at 25 °C and grown for 2 days under a 14/10-h light/dark photo-cycle before treatment initiation. For cold stress, the seedlings were exposed to 4 °C in the dark for 2 h. For salt stress, the seedlings were transferred to a culture medium containing 250 mM NaCl for 24 h. All of the plants were flash frozen in liquid nitrogen immediately after the respective stress treatment and stored at −80 °C prior to RNA extraction.

### Real-time PCR

Total RNA was extracted from the leaves of stress-treated seedlings using TriPure Isolation Reagent (Roche Diagnostics, Indianapolis, IN). The RNA was subsequently treated with Deoxyribonuclease (RT Grade) for Heat Stop (Nippon gene). The DNase-treated RNA was reverse-transcribed using a Transcriptor First Strand cDNA Synthesis Kit (Roche Diagnostics). Real-time PCR was performed using a LightCycler^®^ 1.5 (Roche Diagnostics) real-time instrument with LightCycler^®^ FastStart DNA Master^PLUS^ SYBR Green I (Roche Diagnostics). Samples were amplified as follows: initial denaturation step at 95 °C for 10 min to activate polymerase, followed by 55 cycles of denaturation at 95 °C for 5 s, annealing at a specific temperature (ESM Table 1) for 8 s, and extension at 72 °C for 12 s. *RUBQ* (GenBank Accession No. AK121590) was used as an internal reference gene for calculating the relative transcript levels of the target genes. Expression levels of the target genes were determined using three biological replications. The relative expression levels of target genes were then determined in comparison with those of non-inserted lines under the control condition.

## Results

### Mutation screening of stress tolerance genes with the Gimbozu population

**Fig. 2:**
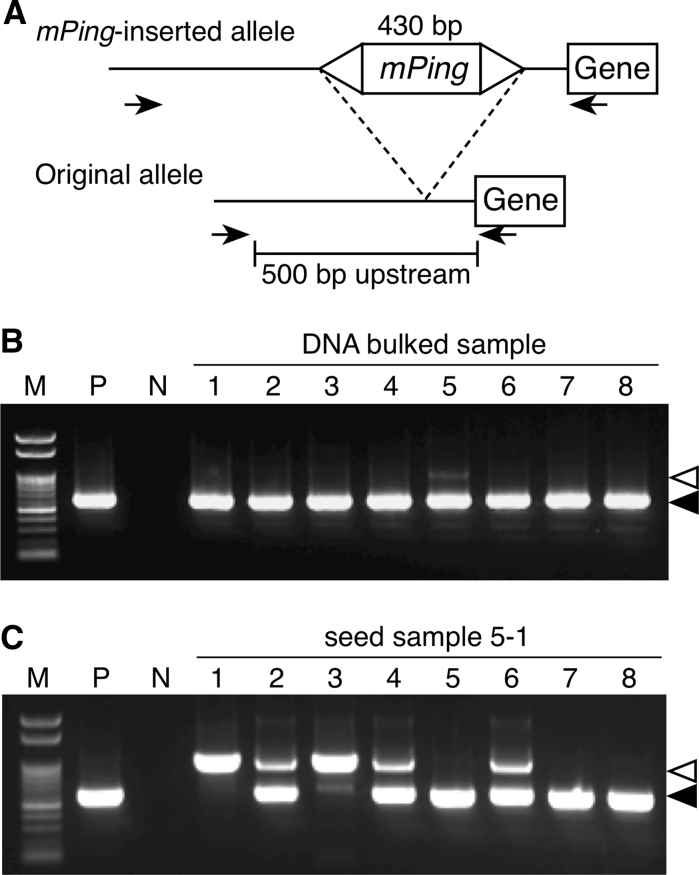
Detection of *mPing*-inserted alleles by PCR. **a** Schematic view of the positions of designed primer pairs. *Arrows* position of site-specific primers. **b** An example of the primary screening by PCR. *mPing* insertion was detected in the No. 5 DNA bulked sample. *Filled arrowhead*, *open arrowhead* original amplicon and the *mPing*-inserted amplicon, respectively. **c** Example of the secondary screening by PCR. Segregation of the *mPing* insertion allele in the selfed progeny of the No. 1 panicle of the No. 5 seed bag (5–1 plant). *M* 100-bp DNA ladder, *P* Nipponbare, *N*, standard D.W

We selected as target genes those stress-related genes whose overexpression could induce high-stress tolerance in rice plants, except for *salT* and *wsi18* (Table 1). *mPing*-inserted promoters were found in *OsDREB1A*, *ZFP252*, *ONAC045*, and *OsCDPK7* (Fig. 2b). For both *OsDREB1A* and *ZFP252*, two *mPing*-inserted promoters were found from two different DNA bulked samples. Sequencing results revealed that two *mPing*-inserted promoters of *OsDREB1A* were identical in terms of the insertion site but the directions of the *mPing* insertion were opposite. This result clearly shows that two *mPing*-inserted promoters of *OsDREB1A* resulted from independent insertion events and that there was an insertion hot spot for *mPing*. Two *mPing*-inserted promoters of *ZFP252* were identical in terms of regards site and direction of *mPing*; however, those may also have resulted from two independent insertion events that occurred at the same insertion hot spot. We used only one of the aforementioned lines for further analysis. The *mPing*-inserted promoters were segregated in seed samples (Fig. 2c), and we selected one *mPing*-inserted promoter homozygous plant and one *mPing*-non-inserted promoter homozygous plant from each segregating line. The progeny lines of those selected plants were named as shown in Table 2.

**Table 2:** Five paired lines originated from a single Gimbozu plant that differ in *mPing* insertion compared to the upstream region of five targeted genes

Gene	Line name	Insertion site from TSS (bp)	Orientation^a^ of *mPing* insertion
*OsDREB1A*	mfDREB+	−224	Forward
	mfDREB−	No	
*OsDREB1A*	mrDREB+	−224	Reverse
	mrDREB−	No	
*ZFP252*	mZFP+	−446	Forward
	mZFP−	No	
*ONAC045*	mNAC+	−263	Reverse
	mNAC−	No	
*OsCDPK7*	mCDPK+	−17	Reverse
	mCDPK−	No	

^a^Forward means that the sense strand of *Ping*’s promoter sequence on *mPing* is forward relative to the target gene; reverse means that the sense strand of *Ping*’s promoter sequence on *mPing* is reverse relative to the target gene

### Database search of *mPing*-inserted promoter

**Fig. 3:**
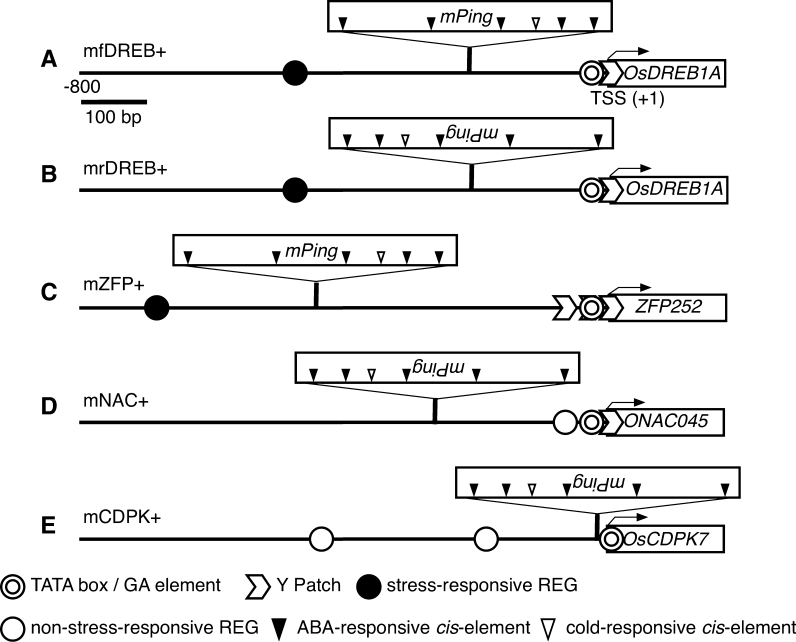
Distribution of putative core promoters in upstream regions of target genes. *Y Patch* Pyrimidine patch, *REG* regulatory element group *ABA* abscisic acid

The *cis*-element interacting with the transcription factors is important for the regulation of gene expression. Sequence comparison between the original promoter and the *mPing*-inserted promoter will help predict and evaluate the effects of *mPing* insertion on the stress response of genes. Using the PPDB (Yamamoto and Obokata 2008), we conducted a database search of the promoter for *OsDREB1A*, *ZFP252*, *ONAC045*, and *OsCDPK7* and estimated that the core promoters consists of the TATA box, Y Patch, and REG. The function feature of REG was searched using PLACE (Higo et al. 1999; Table 3; Fig. 3). In *OsDREB1A*, the TATA box, Y Patch, and REG were mined in the region −38, −10, and −486 bp from the TSS, respectively. The REG contains two etiolation-responsive *cis*-elements (ABRELATERD1 and ACGTATERD1), a calcium-responsive *cis*-element (ABRERATCAL), and three ABA-responsive *cis*-elements (CACGTGMOTIF, EBOXNNAPA, and MYCCONSENSUSAT). In mfDREB+ and mrDREB+, *mPing* insertion was located between the TATA box and the REG. In *ZFP252*, the TATA box (−31 bp) and Y Patch (−17, −39, and −82 bp) were mined. REG was mined at −701 bp from the TSS and contained a gibberellic acid-responsive *cis*-element (WRKY71OS), two etiolation-responsive *cis*-elements (ABRELATERD1 and ACGTATERD1), and two auxin- and salicylic acid-responsive *cis*-elements (ASD1MOTIFCAMV and HEXMOTIFTAH3H4). mZFP+ has a *mPing* insertion between the TATA box and the REG. In *ONAC045*, the TATA box, Y Patch, and a REG-containing photosynthesis-relative *cis*-element (SITEIIATCYTC) were mined at −33, −15, and −85 bp from the TSS, respectively. mNAC+ has an *mPing* insertion −263 bp from the TSS. In *OsCDPK7*, instead of the TATA box, a GA element was mined −17 bp from the TSS as a core promoter (Yamamoto et al. 2009). REGs were mined −222 and −460 bp from the TSS and contained two light-responsive *cis*-elements (CIACADIANLELHC and SITEIIATCYTC) and a calmodulin-related *cis*-element (CGCGBOXAT), respectively. In mCDPK+, the *mPing* insertion was located 1 bp downstream of the GA element.

**Table 3:** Results of database search of promoter region

Gene	Core promoter	Site from TSS (bp)	Name of *cis*-elements in place	Sequence	Function
*OsDREB1A* (AK105599)	TATA box	−27 to −38			
	Y Patch	−1 to −10			
	REG (CCCACGTG)	−479 to −485	ABRELATERD1	ACGTG	Response to etiolation
			ABRERATCAL	MACGYGB	Response to calcium
			ACGTATERD1	ACGT	Response to etiolation
			CACGTGMOTIF	CACGTG	G-box
			EBOXBNNAPA	CANNTG	E-box
			MYCCONSENSUSAT	CANNTG	Response to abscisic acid and cold
*ZFP252* (AY219847)	TATA box	−23 to −31			
	Y Patch	−8 to −17			
		−32 to −39			
		−74 to −82			
	REG (CACGTCAC)	−694 to −701	WRKY71OS	TGAC	Response to gibberellic acid
			ABRELATERD1	ACGTG	Response to etiolation
			ASF1MOTIFCAMV	TGACG	Response to auxin and salicylic acid
			GTGANTG10	GTGA	Pollen-specific
			ACGTATERD1	ACGT	Response to etiolation
			HEXMOTIFTAH3H4	ACGTCA	Rice OBF1-homodimer-binding site
*ONAC045* (AK067922)	TATA	−25 to −33			
	Y Patch	−7 to −15			
	REG (TTGTGGGCTTC)	−75 to −85	SITEIIATCYTC	TGGGCY	Relative to cytochrome, oxidative phosphorylation
*OsCDPK7* (AK061881)	GA	−10 to −17			
	REG (CAAGCCCATCA)	−212 to −222	CIACADIANLELHC	CAANNNNATC	Relative to circadian and response to light
			SITEIIATCYTC	TGGGCY	Relative to cytochrome, oxidative phosphorylation
	REG (CTCGCGCGC)	−452 to −460	CGCGBOXAT	VCGCGB	Response to calmodulin

### Expression changes caused by *mPing*-inserted promoter under cold and salt stress conditions

**Fig. 4:**
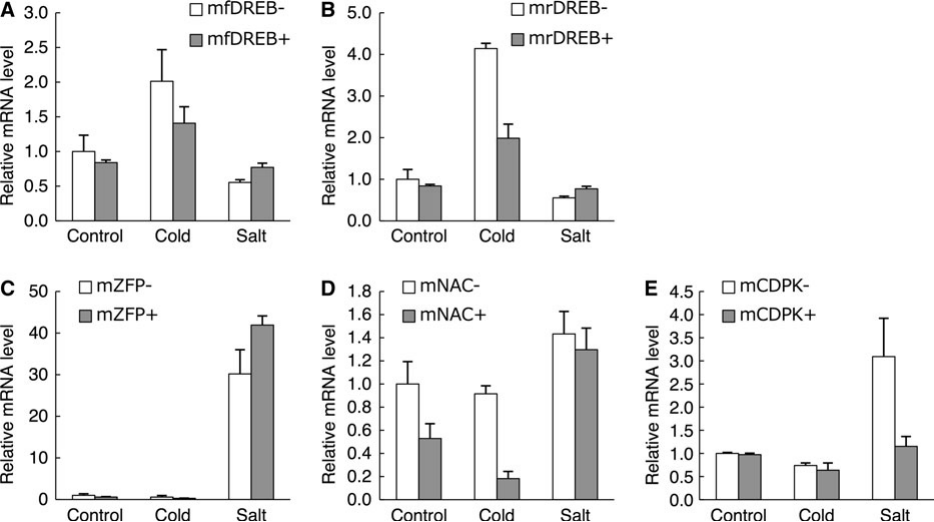
Real-time PCR analysis of *OsDREB1A* (**a**, **b**), *ZFP252* (**c**), *ONAC045* (**d**), and *OsCDPK7* (**e**). Total RNA was extracted from *mPing*-inserted lines and non-inserted lines with no treatment (*Control*), 4 °C for 2 h (*Cold*), and 250 mM NaCl for 24 h (*Salt*). The expression levels of target genes were exhibited as relative values to those of non-inserted lines under the control condition. Data are shown as a the mean ± standard error of three replications

To clarify the impact of *mPing* insertion on the cold and salt response of the neighboring gene expression, we monitored the transcription of *OsDREB1A*, *ZFP252*, *ONAC045*, and *OsCDPK7* under cold and salt stress conditions. The expression levels of the target genes are shown in Fig. 4 as relative values to those of non-inserted lines under the control condition.

The expression level of *OsDREB1A* in mfDREB− and mrDREB− was upregulated under cold stress, but it did not change under salt stress (Fig. 4a, b). In mfDREB+ and mrDREB+, the expression level of *OsDREB1A* was also accelerated only under cold stress. Comparison of mfDREB+ and mrDREB+ revealed that the orientation of *mPing* did not affect the stress response of *OsDREB1A*. Under the normal condition, the expression level of *OsDREB1A* was not affected by the *mPing* insertion, irrespective of orientation. The database search indicated that *mPing* insertion between the TATA box and REG did not significantly affect gene expression under the non-stress condition. The REG of *OsDREB1A* contains a cold-responsive *cis*-element, such as CACGTGMOTIF and MYCCONSENSUSAT. The degree of the cold response of *OsDREB1A* in mfDREB+ and mrDREB+ was less than that in mfDREB− and mrDREB−. This ineffective cold-responsive *cis*-element was caused partly by the increased distance between the REG and TSS due to the *mPing* insertion. The loss of the REG effect could not be fully recovered by the *cis*-element within *mPing*.

In mZFPs, the expression level of *ZFP252* was enhanced under salt treatment but not under cold treatment (Fig. 4c). In mZFP+, the expression level of *ZFP252* under normal conditions was equal to that of mZFP− as the insertion of *mPing* did not change the position of the TATA box or the Y Patch (Fig. 3). In mZFP+, the expression level of *ZFP252* was also increased only under salt stress, and its expression level was higher than that of mZFP−. The database search indicated that the REG of *ZFP252* contained the auxin- and salicylic acid-responsive *cis*-element ASF1MOTIFCAMV. The auxin- and salicylic acid-responsive gene has been found to also respond to salt stress (Shim et al. 2003; Jain and Khurana 2009). In mZFP+, *mPing* insertion changes the distance between the REG and the TSS from −694 to −1,124 bp, but *mPing* insertion provides ASF1MOTIFCAMV as this *cis*-element is also located in *mPing*. This compensation of *cis*-element may one of the reasons why the salt-response in mZFP+ did not change.

In mNAC−, the expression level of *ONAC045* was upregulated under salt stress but not under cold stress. With respect to the effects of *mPing* insertion, the expression level of *ONAC045* did not change under the salt stress condition, but that of mNAC+ was lower than that of mNAC− under normal and cold stress conditions (Fig. 4d). As the database search could not mine stress-responsive *cis*-elements, there must be potential salt-responsive *cis*-elements in the region unaffected by *mPing* insertion.

The expression of *OsCDPK7* was upregulated under the salt stress condition but not under that of cold stress. mCDPK+ exhibited an expression level equal to that of mCDPK-under normal and cold stress conditions, but its expression level under the salt stress condition decreased with *mPing* insertion (Fig. 4e). The *mPing* was inserted without disrupting the GA element and its insertion site is very near the TSS. The effect of the *cis*-element is usually affected by its orientation when it is located near the TSS. In mCDPK+, the direction of the cold-responsive *cis*-element on *mPing* is opposite to that of the TSS, thereby rendering *OsCDPK7* cold-inducible. Since *mPing* insertion reduced the salt response of *OsCDPK7*, the function of an as yet unidentified salt-responsive *cis*-element was disturbed by the insertion.

## Discussion

In earlier studies (Naito et al. 2006, 2009), we showed that *mPing* has a high mobility in rice cv. Gimbozu and that it preferentially inserts into the 5′-flanking region of genes that render adjacent genes stress-inducible. In the present study, we conducted a screening analysis of the *mPing*-inserted promoter with the aim of modifying the expression of stress-inducible genes.

Based on the number of genes in the rice genome and the de novo insertion frequency of *mPing*, we predicted that screening of 11,520 Gimbozu plants would enable us to find at least one *mPing*-inserted promoter in about 78 % of the rice genes. Among the 17 genes selected for targeting, we found five genes with an *mPing*-inserted promoter, which is equivalent to 29 % of the targeted genes. Thus, the number of new insertions of *mPing* was not 40 per plant per generation, but only ten per plant per generation. In this latter case, approximately 100,000 plants should be cultivated to gain an *mPing*-inserted promoter in 96 % of the genes. In our previous study, the rate of de novo insertion was estimated by multiplying the number of de novo insertions observed using the Transposon Display technique with 16 primer sets selected from 64 possible primer sets. This different technology could partly explain the large difference in the de novo insertion frequency of *mPing* between the current and previous studies. We detected two independent *mPing* insertion events at exactly the same site, indicating the presence of a hot spot targeted by *mPing*. The large difference in *mPing* insertion frequency among genes could be one of the main reasons for this inconsistency. Further studies should be conducted to clarify the nature of the hot spot targeted by *mPing*.

We expected that *mPing* insertion would render adjacent genes salt and/or cold stress inducible and would increase the response of stress-tolerance genes under stress conditions. The expression analysis of mZFP+, which has a *mPing* insertion at −446 bp from the TSS of *ZFP252*, promoted the upregulation of *ZFP252* under the salt stress condition. With the exception of mZFP+, the *mPing*-inserted promoter reduced the original stress response of neighboring genes. Our database search revealed that *mPing* insertion relocated the stress-responsive core *cis*-elements, thereby weakening the interaction between *trans*-elements and *cis*-elements. These results indicate that it is difficult to strengthen the response of stress-responsive genes by stress-responsive *cis*-elements in *mPing*. However, they do clarify that the *mPing* insertion could make adjacent non-stress-responsive genes stress responsive by providing de novo stress-responsive *cis*-elements.

Analysis of *cis*-elements revealed that the *mPing* insertion did not disrupt core promoter elements such as the TATA box or GA element. In our previous study (Naito et al. 2009), *mPing* insertions made adjacent genes stress inducible even though their TATA boxes (Os01g0178500, Os02g0582900) were disrupted. Analysis with PPDB and PLACE shows that *mPing* has the TATA box and GA element in the forward strand and the Y Patch and CA element in the reverse strand (ESM Table 3). Therefore, *mPing* may act as a core promoter, even if its insertion disrupts the original TATA box.

Our results clearly show that the expression profile of alleles with an *mPing*-inserted promoter could be altered. *mPing* strengthens the expression of *ZFP252* under salt stress conditions. Since the overexpression of *ZFP252* exhibited high salt stress tolerance compared to the wild type (Xu et al. 2008), mZFP+ could be useful in salt tolerance breeding. In addition, some genes negatively regulate stress tolerance (Magnani et al. 2004; Jeon et al. 2010). It should be possible to gain stress-tolerant plants by screening negative-regulating genes that have an *mPing* insertion in their promoter. Furthermore, it is interesting to note that in most cases the *mPing*-inserted promoter did not affect the expression level under normal conditions. Transgenic plants constitutively expressing the stress tolerance gene grow well under stress conditions; however, a strong constitutive overexpression plant is likely to exhibit poor growth under normal conditions (Kasuga et al. 1999; Hsieh et al. 2002). The use of a stress-inducible promoter could prevent these problems (Nakashima et al. 2007). Thus, *mPing*-inserted promoters have the potential to improve stress tolerance without having negative effects on growth or productivity under normal conditions. In terms of Gimbozu, it is also of note that all of the *mPing*-inserted promoters originated from natural mutation. Thus, it is possible to combine these with a conventional breeding program without any restriction applied to living modified organisms. These advantages of the *mPing*-inserted promoter are useful in altering the gene expression profiles under several kinds of stress conditions. We look forward to screening the *mPing*-inserted promoter on a larger scale to extend the collection of genes with *mPing*-inserted promoters and thereby enable the further selection of genes with modified stress response and use.

## Electronic supplementary material

Below is the link to the electronic supplementary material.

Supplementary material 1 (DOCX 30 kb)
