# Supplementary material for: Utilization of transposable element mPing as a novel genetic tool for modification of the stress response in rice
Source: Mol Breed. 2013 Jun 8;32(3):505–16. doi: 10.1007/s11032-013-9885-1 (PMC3782648; doi:10.1007/s11032-013-9885-1)
Supplement: Supplementary file 1 — Supplementary material 1 (DOCX 30 kb) [file 11032_2013_9885_MOESM1_ESM.docx]

Supplemental Table 1 Primer pairs for screening of *mPing*-inserted promoters

| Gene | Name of primer | | Primer sequence | | Amplified site*  (Product size) (bp) |
| --- | --- | --- | --- | --- | --- |
| *OsDREB1A* | DRE_2up_F | : | | TTCTGCTTGCTCCTGATTCC | -674 ~ +46 |
| (Os09t0522200-01) | DRE_2up_R | : | | ACTTCCAGGCGAAAAATGAC | (720) |
| *DREB1D* | DRE_4up_F | : | | GTGGCTAGGACTTGAAGATGCTG | -895 ~ +154 |
| (Os06t0165600-01) | DRE_4up_R | : | | GCACCCCACGGAACACTAGGTGC | (1049) |
| *OsDREB1F* | DRE_8up_F | : | | CACAACTAATGCTCGATGTCCAAA | -519 ~ +81 |
| (Os01t0968800-00) | DRE_8up_R | : | | CTCGGTGTCCATGGTCGAA | (600) |
| *ZFP252* | ZFP_1up_F | : | | GGGAAAGAGAAGTGCACAAGAAA | -523 ~ +71 |
| (Os12t0583700-00) | ZFP_1up_R | : | | CAATTAGCTCCTCTACACCAACACA | (594) |
| *ZFP182* | ZFP_2up_F | : | | CCGCGATCATAGACACAATCC | -462 ~ +95 |
| (Os03t0820300-01) | ZFP_2up_R | : | | TGCCTCGGGTGCTTCATCT | (557) |
| *SNAC1* | NAC_1up_F | : | | TTTCCCCTTTTCGCTCCAC | -529 ~ +87 |
| (Os03t0815100-01) | NAC_1up_R | : | | ATCCCCATCGCTTCTTGCT | (616) |
| *OsNAC6* | NAC_2up_F | : | | CGAGCTCGCTACTACTACTGCTCT | -440 ~ +72 |
| (Os01t0884300-01) | NAC_2up_R | : | | CGCATCCTTATCCCACCAC | (512) |
| *ONAC045* | NAC_3up_F | : | | GAATAGACAAGCGCCTAGCTGAA | -540 ~ +52 |
| (Os11t0127600-01) | NAC_3up_R | : | | AAGCAACAAAGGTGGAGACGA | (592) |
| *OsLEA3-1* | LEA_1up_F | : | | AGCGAAAGGTAGCAGAACACATC | -531 ~ +121 |
| (Os05t0542500-01) | LEA_1up_R | : | | GTCCTGGTGGGAAGCCATT | (652) |
| *MYBS3* | MYB_1up_F | : | | TGGAGACGGAGATGGTGGT | -485 ~ +64 |
| (Os10t0561400-02) | MYB_1up_R | : | | GTTCACCCGTGGCACATTAG | (549) |
| *OsNHX1* | NHX_1up_F | : | | AAACTGCCTTTGAACCCTAGCA | -420 ~ +138 |
| (Os07t0666900-1) | NHX_1up_R | : | | ACGAACAGATTGAAACGAGGAAC | (558) |
| *OsGS2* | GS_1up_F | : | | AGGCATTGCACGGACTCAC | -476 ~ +64 |
| (Os04t0659100-01) | GS_1up_R | : | | ACGTTTCTAAACCGCTAATCTCCTC | (540) |
| *OsCDPK7* | CDPK_1up_F | : | | GGGGCAACGAGACGAAA | -324 ~ +132 |
| (Os04t0584600-02) | CDPK_1up_R | : | | CGAGAGGGAACTGAACTGGA | (456) |
| *SalT1* | SalT_1up_F | : | | GCAGAGCTAGCTAGAACAACATCAA | -390 ~ +114 |
| (Os01t0348900-01) | SalT_1up_R | : | | GTCCCTAAATCGCCAGAAGATAAG | (504) |
| *wsi18* | wsi_1up_F | : | | GAATCACTCACCAACACACGAA | -442 ~ +115 |
| (Os01t0705200-01) | wsi_1up_R | : | | AAGCAAAGAAAGCACAGCACA | (557) |
| *OsWRKY11* | WRKY_1up_F | : | | GCATGCTATGCCCAATATATACCA | -488 ~ +132 |
| (Os01t0626400-01) | WRKY_1up_R | : | | TGTACACGTAATCAGCGCCTTC | (620) |
| *OsbZIP* | ZIP_1up_F | : | | ATGTGGAGCTCTGGCGTTG | -507 ~ +67 |
| (Os02t0766700-01) | ZIP_1up_R | : | | GATTGCCTGGTTGGTGGTG | (574) |

* The position is indicated in relative to the TSS.

Supplemental Table 2 Primers for real-time PCR

| Gene | Name of primer | | Primer sequence | Product size (bp) |
| --- | --- | --- | --- | --- |
| *OsDREB1A* | DRE_2RT_F | : | GGACCTGTACTACGCGAGCTT | 80 |
|  | DRE_2RT_R | : | GGCAAAATTGTACAGTTGATTGA |  |
| *ZFP252* | ZFP_1RT_F | : | TCACCACTCCTCTTCTCCATTTC | 194 |
|  | ZFP_1RT_R | : | CGCCTCCTCCTCACTACTACTTCT |  |
| *ONAC045* | NAC_3RT_F | : | TTGCCAATCGGCGAGGT | 143 |
|  | NAC_3RT_R | : | TTGGTGGCTCGGTTGGTT |  |
| *OsCDPK7* | CDPK_1RT_F | : | GGGACCTCAAGCCAGAAAAC | 136 |
|  | CDPK_1RT_R | : | CGTAATATGGGCTTCCGACAA |  |
| *RUBQ* | RUBQ_F | : | GCTGCTGTTCTTGGGTTCACA | 150 |
| (Os06t0681400-01) | RUBQ_R | : | CGTTTCAGACACCATCAAACCA |  |

Supplemental Table 3 Results of searching for the core promoter element

(except for REGs) on *mPing* with ppdb and PLACE

| element | site | strand |
| --- | --- | --- |
| CA element | 303 bp | reverse |
| GA element | 93 bp | forward |
| TATA box | 72 bp | forward |
| Y patch | 102, 378 bp | reverse |
